# Chronic Obstructive Pulmonary Disease Prevalence Among Adults Who Have Never Smoked, by Industry and Occupation — United States, 2013–2017

**DOI:** 10.15585/mmwr.mm6813a2

**Published:** 2019-04-05

**Authors:** Girija Syamlal, Brent Doney, Jacek M. Mazurek

**Affiliations:** 1 Respiratory Health Division, National Institute for Occupational Safety and Health, CDC, Morgantown, West Virginia.

## Abstract

Tobacco smoking is a major risk factor for chronic obstructive pulmonary disease (COPD), a debilitating respiratory condition with high mortality and morbidity (*1*,*2*). However, an estimated 24% of adults with COPD have never smoked (*3*,*4*). Among these persons, 26%–53% of COPD can be attributed to workplace exposures, including dust, fumes, gases, vapors, and secondhand smoke exposure (*4*–*6*). To assess industry-specific and occupation-specific COPD prevalence among adults aged ≥18 years who have never smoked and who were employed any time during the past 12 months, CDC analyzed 2013–2017 National Health Interview Survey (NHIS) data. Among an estimated 106 million workers who had never smoked, 2.2% (2.4 million) have COPD. Highest prevalences were among workers aged ≥65 years (4.6%), women (3.0%), and those reporting fair/poor health (6.7%). Among industries and occupations, the highest COPD prevalences were among workers in the information industry (3.3%) and office and administrative support occupations (3.3%). Among women, the highest prevalences were among those employed in the information industry (5.1%) and in the transportation and material moving occupation (4.5%), and among men, among those employed in the agriculture, forestry, fishing, and hunting industry (2.3%) and the administrative and support, waste management, and remediation services industry (2.3%). High COPD prevalences in certain industries and occupations among persons who have never smoked underscore the importance of continued surveillance, early identification of COPD, and reduction or elimination of COPD-associated risk factors, such as the reduction of workplace exposures to dust, vapors, fumes, chemicals, and exposure to indoor and outdoor air pollutants.

NHIS data are collected annually from a nationally representative sample of the civilian noninstitutionalized U.S. population through a personal interview. To improve the precision and reliability of estimates, data collected during 2013–2017 were combined. Survey response rates ranged from 61.2% in 2013 to 53.0% in 2017.* Respondents were considered to be employed if they were “working for pay at a job or business,” or “with a job or business but not at work,” or “working, but not for pay, at a family-owned job or business” any time during the 12 months preceding the interview. Information on participants’ current industry and occupation was categorized into 21 industry groups and 23 occupation groups.^†^ Participants with COPD were identified by a positive response to any of the following three questions: 1) “Have you ever been told by a doctor or other health professional that you had chronic obstructive pulmonary disease, also called COPD?”; 2) “Have you ever been told by a doctor or other health professional that you had emphysema?”; or 3) “During the past 12 months, have you been told by a doctor or other health professional that you had chronic bronchitis?” Persons were considered to have never smoked if they had never smoked or smoked <100 cigarettes during their lifetime. Persons were considered to have ever smoked if they had smoked >100 cigarettes during their lifetime and includes both current cigarette smokers and former cigarette smokers. Respondent self-reported health status at the time of interview and the number of physician office visits, emergency department (ED) visits, and lost work days in the past 12 months resulting from any illness or injury were assessed.^§^

Data were analyzed using SAS (version 9.4; SAS Institute) and were adjusted for nonresponse and weighted to be nationally representative, and variance estimates were calculated to account for the clustered survey design. The proportions of workers who reported emphysema, chronic bronchitis, and COPD diagnosis were assessed separately among those who never smoked and those who ever smoked. Prevalence estimates with relative standard error (standard error of the estimate divided by the estimate) ≥30% were not reported. Participants with unknown or missing information for COPD were excluded from the analysis. Two-sided t-tests were used to determine statistically significant (p<0.05) differences between point estimates.

During 2013–2017, among an estimated 164 million U.S. adults aged ≥18 years who were working any time during the 12 months preceding the interview, COPD prevalences were 6.0% (3.4 million) among those who ever smoked and 2.2% (2.4 million) among those who never smoked. The proportions of workers who reported an emphysema, chronic bronchitis, or COPD diagnosis were 1.1%, 3.8%, 2.5%, respectively for those who ever smoked (35% of workers), and 0.1%, 1.9%, and 0.4%, respectively for those who never smoked (65% of workers). 

Among workers who never smoked, the highest COPD prevalences were among women (3.0%), adults aged ≥65 years (4.6%), and those reporting fair/poor health (6.7%), more than three physician office visits in past 12 months (4.2%), more than three ED visits in past 12 months (10.3%), and >7 days of work lost because of any illness or injury (6.6%). By sex, race, and ethnicity, COPD prevalences were highest among non-Hispanic white men (1.7%) and non-Hispanic black women (3.7%) (Table 1). Among persons who never smoked, those with COPD missed an average of 14.9 work days (15.6 days for women and 13.6 days for men) because of any illness or injury compared with persons who did not have COPD, who missed an average of 5.4 work days (6.4 days for women and 4.4 days for men).

**TABLE 1:** Prevalence of chronic obstructive pulmonary disease (COPD)* among employed persons who never smoked, by sex and selected characteristics — National Health Interview Survey, United States, 2013–2017

Characteristic	Men		Women		All workers
	Employed adults^†^ (x 1,000)	% (95% CI)	Employed adults^†^ (x 1,000)	% (95% CI)	% (95% CI)
**Total**	**52,084**	**1.5 (1.3–1.7)**	**54,075**	**3.0 (2.7–3.2)**	**2.2 (2.1–2.4)**
**Age group (yrs)**					
18–24	8,670	1.2 (0.7–1.8)	8,978	2.1 (1.5–2.8)	1.7 (1.3–2.1)
25–44	22,662	0.9 (0.7–1.1)	23,252	2.2 (1.9–2.5)	1.6 (1.4–1.8)
45–64	18,401	2.0 (1.7–2.4)	18,992	3.9 (3.5–4.3)	3.0 (2.7–3.3)
≥65	2,351	3.9 (2.7–5.1)	2,853	5.2 (4.1–6.3)	4.6 (3.8–5.4)
**Race/Ethnicity**					
Hispanic	10,196	1.3 (1.0–1.7)	9,643	2.1 (1.7–2.6)	1.7 (1.4–2.0)
White, non-Hispanic	31,921	1.7 (1.4–1.9)	31,864	3.2 (2.9–3.5)	2.5 (2.2–2.7)
Black, non-Hispanic	6,175	1.2 (0.7–1.6)	7,989	3.7 (3.1–4.4)	2.6 (2.2–3.0)
Other	3,793	1.1 (0.5–1.7)	4,580	1.6 (1.1–2.1)	1.3 (1.0–1.7)
**Education**					
≤High school/GED	15,273	1.7 (1.4–2.0)	13,681	3.3 (2.8–3.8)	2.5 (2.2–2.7)
>High school	36,646	1.4 (1.2–1.6)	40,260	2.9 (2.6–3.1)	2.2 (2.0–2.3)
Unknown	165	—^§^	135	—	—
**Poverty status^¶^**					
Poor	3,902	1.5 (1.0–2.0)	5,122	3.1 (2.4–3.7)	2.4 (2.0–2.8)
Near poor	6,460	1.8 (1.3–2.3)	7,695	3.6 (2.9–4.3)	2.8 (2.3–3.2)
Not poor	38,474	1.5 (1.3–1.7)	37,858	2.8 (2.5–3.0)	2.1 (2.0–2.3)
Unknown	3,249	1.0 (0.5–1.5)	3,401	3.3 (2.4–4.3)	2.2 (1.6–2.8)
**Health insurance status**					
Not insured	6,754	1.2 (0.8–1.6)	5,547	2.9 (2.1–3.8)	2.0 (1.6–2.4)
Insured	45,006	1.5 (1.3–1.7)	48,211	3.0 (2.7–3.2)	2.3 (2.1–2.4)
Unknown	324	—	317	—	—
U.S. Census region**					
Northeast	9,284	1.6 (1.1–2.1)	9,480	2.3 (1.8–2.8)	2.0 (1.6–2.3)
Midwest	11,406	1.6 (1.2–2.0)	11,656	3.3 (2.7–3.8)	2.5 (2.1–2.8)
South	18,205	1.4 (1.2–1.7)	19,759	3.2 (2.9–3.6)	2.4 (2.2–2.6)
West	13,189	1.4 (1.1–1.8)	13,180	2.7 (2.3–3.2)	2.1 (1.8–2.4)
**Self–rated health^††^**					
Excellent/Very good/Good	49,697	1.3 (1.2–1.5)	51,142	2.7 (2.5–2.9)	2.0 (1.9–2.2)
Poor/Fair	2,379	5.3 (3.9–6.7)	2,909	7.8 (6.4–9.2)	6.7 (5.7–7.7)
**Physician office visits^§§^**					
None	13,632	0.8 (0.5–1.1)	7,058	1.4 (0.8–2.0)	1.0 (0.7–1.3)
1–3	27,135	1.2 (1.0–1.4)	26,795	2.2 (1.9–2.5)	1.7 (1.5–1.9)
>3	10,671	3.3 (2.7–3.8)	19,566	4.7 (4.2–5.1)	4.2 (3.8–4.5)
**Emergency department visits^¶¶^**					
None	45,561	1.2 (1.0–1.4)	44,728	2.4 (2.1–2.5)	4.1 (3.8–4.4)
1–3	5,697	3.4 (2.6–4.2)	8,122	5.7 (4.9–6.5)	4.8 (4.2–5.4)
>3	268	12.1 (6.1–18.1)	697	9.6 (6.1–13.1)	10.3 (7.3–13.3)
Lost work days***					
None	32,497	1.1 (0.9–1.3)	30,264	2.0 (1.8–2.3)	1.5 (1.4–1.7)
1–7	16,771	1.8 (1.5–2.2)	20,074	3.4 (3.0–3.8)	2.7 (2.4–3.0)
>7	2,692	4.5 (3.3–5.8)	3,532	2.0 (1.8–2.3)	6.6 (5.7–7.5)

**Abbreviations:** CI = confidence interval; GED = General Educational Development certificate.

* Proportion of workers who positively answered the question “Have you ever been told by a doctor or other health professional that you had chronic obstructive pulmonary disease, also called COPD?” Proportion of workers who positively answered the question “Have you ever been told by a doctor or other health professional that you had emphysema?” Proportion of workers who were solely identified as having COPD by a positive answer to the question “During the past 12 months, have you been told by a doctor or other health professional that you had chronic bronchitis?” For this report survey respondents with COPD were those who positively answered to any of these three questions.

^†^ Adults who reported “working at a job or business”; “with a job or business but not at work”; or “working, but not for pay, at a family–owned job or business” during the last 12 months of the survey interview and have never smoked. Estimates are weighted to provide national estimates for current employment and are presented in thousands.

^§^ Dashes indicate estimates suppressed because relative standard error for the estimate was ≥30%.

^¶^ Poverty status is based on family income and family size using the U.S. Census Bureau's poverty thresholds for the previous calendar year. In National Health Interview Survey, “poor” persons are defined as having incomes below the federal poverty level (FPL), “near poor” are defined as having incomes of 100% to <200% of the FPL, and “not poor” are defined as having incomes that are ≥200% of the FPL. https://www.cdc.gov/nchs/data/nhis/SHS_Tech_Notes.pdf.

** https://www2.census.gov/geo/pdfs/maps–data/maps/reference/us_regdiv.pdf.

^††^ Based on the response to “Would you say your health in general is excellent, very good, good, fair, or poor?”

^§§^ Based on the response to “During the past 12 months, how many times have you seen a doctor or other health care professional about your own health at a doctor’s office, a clinic, or some other place?”

^¶¶^ Based on the response to “During the past 12 months, how many times have you gone to a hospital emergency room about your own health (This includes emergency room visits that resulted in a hospital admission.)?”

*** Based on the response to “During the past 12 months, about how many days did you miss work at a job or business because of illness or injury (do not include maternity leave)?”

Among workers who never smoked, COPD prevalences exceeded 3.0% among those in the information (3.3%) and mining (3.1%) industries and in the office and administrative support occupation (3.3%) (Table 2). Sex differences in COPD prevalence were observed by industry and occupation. Among men, the highest COPD prevalences were among those employed in agriculture, forestry, fishing, and hunting (2.3%), the administrative and support, waste management, and remediation services (2.3%), the arts, entertainment, and recreation (2.3%) industries, and protective services occupation (2.3%); among women, the highest prevalences were among those employed in the information industry (5.1%) and transportation and material moving occupation (4.5%).

**TABLE 2:** Prevalence of chronic obstructive pulmonary disease (COPD)* among employed persons who never smoked by sex, industry, and occupation — National Health Interview Survey, United States, 2013–2017

Industry/Occupation	Men		Women		All workers
	No.^†^ x1,000	% (95% CI)	No.^†^ x1,000	% (95% CI)	% (95% CI)
**Industry groups**					
Agriculture, forestry, fishing, and hunting	1,068	2.3 (1.3–3.3)	459	—^§^	2.4 (1.4–3.3)
Mining	408	—	92	—	3.1 (0.4–5.9)
Utilities	666	—	204	—	1.6 (0.4–2.9)
Construction	4,811	1.2 (0.7–1.7)	577	3.9 (1.7–6.1)	1.5 (1.0–2.0)
Manufacturing	6,247	1.3 (0.8–1.8)	3,234	3.4 (2.0–4.7)	2.0 (1.5–2.6)
Wholesale trade	1,502	1.7 (0.7–2.8)	854	4.6 (2.5–6.7)	2.8 (1.7–3.8)
Retail trade	5,027	1.0 (0.7–1.4)	5,844	2.8 (2.1–3.6)	2.0 (1.6–2.5)
Transportation and warehousing	2,841	1.4 (0.9–2.0)	1,011	4.3 (2.6–5.9)	2.2 (1.6–2.8)
Information	1,244	1.9 (0.6–3.3)	917	5.1 (2.8–7.3)	3.3 (2.0–4.5)
Finance and insurance	2,249	1.7 (0.7–2.8)	2,923	3.4 (2.4–4.4)	2.7 (2.0–3.4)
Real estate and rental and leasing	1,050	—	924	3.3 (1.6–5.1)	1.7 (0.8–2.5)
Professional, scientific, and technical services	4,977	1.5 (0.9–2.0)	3,787	1.9 (1.2–2.5)	1.6 (1.2–2.1)
Management of companies and enterprises	27	—	35	—	—
Administrative and support, waste management, and remediation services	2,448	2.3 (1.4–3.2)	1,832	3.3 (2.2–4.4)	2.7 (2.0–3.4)
Education services	3,825	1.9 (1.0–2.8)	8,067	3.3 (2.7–3.8)	2.8 (2.3–3.3)
Health care and social assistance	3,042	1.8 (0.9–2.7)	12,046	2.8 (2.3–3.3)	2.6 (2.2–3.0)
Arts, entertainment, and recreation	1,369	2.3 (0.9–3.7)	1,185	2.6 (1.4–3.9)	2.5 (1.5–3.4)
Accommodation and food services	2,977	1.7 (0.9–2.5)	3,937	2.2 (1.6–2.8)	2.0 (1.5–2.5)
Other services (except public administration)	2,241	1.1 (0.5–1.6)	2,837	2.8 (1.9–3.7)	2.0 (1.5–2.6)
Public administration	2,893	1.5 (0.8–2.2)	2,376	3.4 (2.3–4.5)	2.3 (1.7–3.0)
Armed forces	133	—	33	—	—
Refused, not ascertained, don’t know	1,040	—	901	—	—
**Occupation groups**					
Management	2,054	1.5 (0.9–2.0)	4,347	2.6 (1.9–3.3)	2.0 (1.5–2.4)
Business and financial operations	1,257	1.8 (0.8–2.8)	3,305	2.6 (1.8–3.5)	2.3 (1.6–2.9)
Computer and mathematical	371	1.7 (0.9–2.5)	1,128	2.3 (0.9–3.6)	1.9 (1.1–2.6)
Architecture and engineering	131	1.1 (0.5–1.7)	410	1.4 (0.2–2.6)	1.2 (0.6–1.7)
Life, physical, and social science	179	—	611	2.8 (1.0–4.7)	2.0 (0.9–3.1)
Community and social services	531	—	1,402	3.0 (1.8–4.3)	2.5 (1.5–3.4)
Legal	268	—	680	—	1.8 (0.6–3.0)
Education, training, and library	1,639	2.2 (0.9–3.4)	5,877	3.1 (2.5–3.8)	2.9 (2.3–3.5)
Arts, design, entertainment, sports, and media	467	—	1,212	3.0 (1.7–4.4)	1.7 (1.0–2.4)
Health care practitioners and technical	1,795	—	5,024	2.0 (1.5–2.6)	1.9 (1.4–2.4)
Health care support	1,111	—	2,274	2.7 (1.7–3.8)	2.7 (1.7–3.7)
Protective service	226	2.3 (1.2–3.4)	505	—	2.4 (1.4–3.4)
Food preparation and serving related	1,771	1.7 (0.7–2.6)	3,176	2.4 (1.6–3.2)	2.1 (1.5–2.7)
Building and grounds cleaning and maintenance	963	2.0 (1.1–2.9)	1,835	2.9 (1.8–3.9)	2.4 (1.7–3.1)
Personal care and service	1,396	—	3,099	3.3 (2.4–4.3)	2.8 (2.0–3.6)
Sales and related	2,603	1.0 (0.5–1.4)	5,635	2.5 (1.8–3.3)	1.8 (1.4–2.2)
Office and administrative support	4,762	1.5 (1.0–2.1)	9,218	4.0 (3.3–4.6)	3.3 (2.8–3.8)
Farming, fishing, and forestry	88	2.1 (0.7–3.5)	301	—	1.8 (0.7–2.9)
Construction and extraction	125	1.6 (0.9–2.2)	139	—	1.6 (1.0–2.2)
Installation, maintenance, and repair	81	1.7 (0.8–2.6)	129	—	1.8 (0.9–2.7)
Production	950	1.0 (0.5–1.5)	1,823	4.4 (2.0–6.8)	2.2 (1.3–3.1)
Transportation and material moving	645	1.9 (1.2–2.6)	1,037	4.5 (2.1–6.8)	2.4 (1.7–3.1)
Military	14	—	37	—	—
Refused, not ascertained, don’t know	246	—	901	—	1.1 (0.4–1.7)

**Abbreviation:** CI = confidence interval.

* Proportion of workers who positively answered the question “Have you ever been told by a doctor or other health professional that you had chronic obstructive pulmonary disease, also called COPD?” Proportion of workers who positively answered the question “Have you ever been told by a doctor or other health professional that you had emphysema?” Proportion of workers who were solely identified as having COPD by a positive answer to the question “During the past 12 months, have you been told by a doctor or other health professional that you had chronic bronchitis?” For this report survey respondents with COPD were those who positively answered to any of these three questions.

^†^ Adults who reported “working at a job or business”; “with a job or business but not at work”; or “working, but not for pay, at a family–owned job or business” during the last 12 months of the survey interview and have never smoked. Estimates are weighted to provide national annual estimates for current employment and is presented in thousands.

^§^ Dashes indicate estimates suppressed because relative standard error for the estimate was ≥30%.

## Discussion

An estimated 24% of U.S adults with COPD have never smoked (*3*,*4*). Among persons who never smoked, an estimated 26%–53% of COPD can be attributed to occupational exposures (*4*–*6*). Previous studies have shown that occupational exposures to dust and toxins, as well as biologic and social differences, and genetic factors were associated with increased risk for COPD among persons who never smoked (*1*,*3*,*5*,*7*). Therefore, identifying occupational risk factors is needed for preventing and reducing COPD among workers. This study, which provides industry- and occupation-specific COPD prevalence estimates among 106 million persons who never smoked and were employed any time in the past 12 months, found that two thirds of those with COPD were women. Women who had never smoked had higher COPD prevalences than did men regardless of their sociodemographic characteristics. Within-group variations were observed among sex, race, and ethnicity, with the highest prevalences among non-Hispanic black women and non-Hispanic white men.

National surveys have shown that exposure to vapors, gas, dust, fumes, grain dust, organic dust, inorganic dust, ammonia, hydrogen sulfide, diesel exhaust, environmental tobacco smoke, and chemicals increases the risk for COPD morbidity and mortality among persons who have never smoked (*4*–*6*). For example, exposure to coal mine dust or respirable crystalline silica among workers in the mining industry has been associated with COPD and other pulmonary diseases.^¶^ In this study, office and administrative support workers (including secretaries, administrative and dental assistants, and clerks), protective service workers, and information industry workers (including publishing, telecommunications, broadcasting, and data processing workers) had the highest COPD prevalences. Workers in these industries can be exposed to organic and inorganic dusts, isocyanates, irritant gases, paper dust and fumes from photocopiers, chemicals, oil-based ink, paints, glues, isocyanates, toxic metals, and solvents, all of which are known respiratory irritants and have been associated with bronchitis, emphysema, and COPD.**^,^^††^ In addition, workplace exposures to environmental tobacco smoke can be associated with COPD (*8*).

In this report, although the pattern of responses to all three COPD-related questions among those who ever smoked and those who never smoked was similar (i.e., highest proportions with COPD were among those who were diagnosed with chronic bronchitis), chronic bronchitis was 19 times more frequently reported than emphysema among those who never smoked, compared with 3.5 times among those who ever smoked. These results are similar to those previously reported that a substantial proportion of COPD among the nonsmoker population might be explained by chronic bronchitis (*9*). 

The findings in this report are subject to at least five limitations. First, information on COPD was self-reported and not validated by medical records review or pulmonary function tests. Second, no work history, secondhand smoke exposure, or workplace exposure information was available to assess associations with COPD. Third, only workers employed at some time in the past 12 months were included in this study. Those with severe COPD might have left the workforce, and COPD prevalence might be underestimated. Fourth, despite combining data for multiple years, small sample sizes in certain groups resulted in unreliable estimates. Finally, the survey collected information on any physician office visits or ED visits in the past 12 months and lost workdays because of any illness or injury, and these visits might not be associated with COPD.

The findings of high COPD prevalences among workers who never smoked corroborates findings that occupational exposures, in addition to smoking, might be associated with development of COPD. Higher COPD prevalences in certain industries and occupations underscore the importance of continued surveillance, identification of potential workplace exposures, collection of detailed occupational history, performance of pulmonary function testing, and assessment of environmental tobacco smoke exposure for early diagnosis and treatment of COPD among workers (*10*). Efforts to reduce adverse workplace exposures (including exposure to dust, vapors, fumes, chemicals, and indoor and outdoor air pollutants) and promote research to characterize the many contributing risk factors in COPD are needed to reduce the prevalence of COPD.^§§^

**Summary**

**What is already known about this topic?**

Approximately 25% of adults with chronic obstructive pulmonary disease (COPD) have never smoked, and workplace exposures likely contribute to much of their disease.

**What is added by this report?**

During 2013–2017, an estimated 2.4 million (2.2%) U.S. working adults aged ≥18 years who never smoked had COPD. The highest COPD prevalences among persons who never smoked were in the information (3.3%) and mining (3.1%) industries and office and administrative support occupation workers (3.3%). Women had higher COPD prevalences than did men.

**What are the implications for public health practice?**

Efforts to reduce adverse workplace exposures and promote research to characterize the many contributing risk factors for COPD are needed to improve efforts to prevent and reduce risk for COPD among nonsmoking workers.

## Footnotes

* ftp://ftp.cdc.gov/pub/Health_Statistics/NCHS/Dataset_Documentation/NHIS/2017/srvydesc.pdf.

† ftp://ftp.cdc.gov/pub/Health_Statistics/NCHS/Dataset_Documentation/NHIS/2017/samadult_layout.pdf.

§
ftp://ftp.cdc.gov/pub/Health_Statistics/NCHS/Survey_Questionnaires/NHIS/2017/english/qadult.pdf .

¶
https://www.cdc.gov/niosh/docs/2011-172/pdfs/2011-172.pdf .

**
http://www.ilocis.org/en/contilo.html .

††
https://www.labour.gov.hk/text_alternative/pdf/eng/GN_Printing.pdf .

§§
https://www.nhlbi.nih.gov/health-topics/education-and-awareness/COPD-national-action-plan .
